# Effect of a Strategic Physical Activity Program on Cognitive Flexibility among Children with Internet Addiction: A Pilot Study

**DOI:** 10.3390/children9060798

**Published:** 2022-05-29

**Authors:** Yu-Hsien Tseng, Hsiao-Han Chao, Chiao-Ling Hung

**Affiliations:** 1 Department of Athletics, National Taiwan University, Taipei 10617, Taiwan; tsengblue@ntu.edu.tw (Y.-H.T.); nikkichao@ntu.edu.tw (H.-H.C.); 2 Masters in Sport Facility Management and Health Promotion, National Taiwan University, Taipei 10617, Taiwan

**Keywords:** executive control, switch cost, motor competence, open-skill, cognition

## Abstract

The purpose of this study was to explore whether a strategic physical activity program can improve cognitive flexibility among children with Internet addiction. Ten school-aged children were recruited by distributing flyers at an elementary school in Taiwan. The participants were screened using the Chinese Internet Addiction Scale. Their executive functions were assessed by a task-switching paradigm and their motor competence was evaluated by the Movement Assessment Battery for Children–Second Edition (MABC-2) before and after a 12-week strategic physical activity intervention (twice per week, 90 min per session). The posttest scores showed significant improvements in accuracy in the pure, mixed, and switch trials and in the manual dexterity and total score of the MABC-2 compared with the pretest scores. Despite the inherited limitations of a single group pretest-posttest design this study employed, the findings shed light on the possibility that a strategic physical activity intervention might be a feasible and effective behavioral approach to enhance cognitive function and motor competence in children with Internet addiction. Further studies including a control group, preferably with a randomized controlled trial design, will be needed to validate the findings.

## 1. Introduction

Internet addiction (IA), including addiction to online games and personal computers, has become a widespread phenomenon and a serious public health issue. It has recently been considered for inclusion in the DSM-V [1]. Studies from various parts of Asia suggest that the overall prevalence of IA is approximately 12% among younger people [2], whereas the prevalence rate among school students in Taiwan ranges from 10% to 17.4% [3,4]. IA is associated with numerous negative outcomes, such as lower quality of life, worse scholastic outcomes, and occupational difficulties [5].

The psychopathological foundation of IA is still controversial, and treatment options are generally in the early stages of development. Although psychological and pharmacological interventions have been recommended for improving IA [6], their effectiveness is often reduced by potential side effects and excessive time consumption. Improvement of individuals’ executive functions (EF) is a promising approach to amelioration of the symptoms of IA. Neuropsychological investigations have demonstrated that certain symptoms of IA are related to prefrontal functions, particularly EFs; these include multiple top-down mental processes, such as inhibition, working memory, and cognitive flexibility, that are involved in goal-directed behaviors [7]. This is in line with recent theoretical models on the development and maintenance of the addictive use of the Internet [8]. Furthermore, recent studies have suggested that individuals with IA may exhibit impaired EF, such as attentional inhibition, motor inhibition (and prepotent motor inhibition), decision-making, and working memory [9]. Moreover, an fMRI study suggested that cognitive flexibility was impaired in subjects with IA [10]. These findings point to the potential of improving EFs as a treatment for IA addiction.

Among interventions for improving EF, physical activity (PA) has attracted increased attention. A growing body of evidence suggests that it has positive effects on cognitive function. For example, PA has been found to have a significant and positive impact on cognition in typically developing children [11] and in children with ADHD [12,13]. Another empirical study demonstrated the positive effect of physical exercise on higher-order EF [14]. PA may benefit EF by adjusting relevant brain structures and neurotransmitters, and thus it could serve as an alternative treatment for children with IA. In addition, the past study showed low PA with high screen-based sedentary behavior was 53.7% within IA [15]. Intervention programs like sedentary behavior interventions and maintaining enough PA might contribute to reducing sedentary time [16]. Therefore, designing a PA program that is effective for improving EFs in children with IA is therefore warranted.

Cognitively engaging PA has been suggested to be particularly effective for improving EF. A neuroimaging study showed that performing complex motor tasks co-activated brain regions such as the prefrontal cortex, cerebellum, and basal ganglia, which are related to higher-order cognition [17]. A systematic review also suggested that combining physical exercise with cognitive stimulation may be an effective strategy for improving EF [18]. Among different types of PA, basketball may have good potential for improving EF in children with IA because it demands the timely processing and updating of information related to one’s position on the court and those of one’s teammates, opponents, and the ball simultaneously [19]. Previous studies have shown a positive association between childhood EF and motor competence, a health-related factor that represents the ability to perform complex motor movements (e.g., ball skills, dribbling, kicking) and involves multiple complex cognitive operations such as sequencing, monitoring, and planning [20], in children with ADHD [21]. Moreover, effective performance also requires communication, cooperation, and teamwork. As a team sport in which each player has a specific task according to their position on the court, it also requires attention while playing with teammates and opponents [22]. Previous studies have found that the motor competence of young basketball players was positively correlated with cognition (a denomination task and a visuo-spatial working memory task) and motor manual sequencing (ball skills and manual dexterity tasks) [23,24]. As such, basketball could be a good candidate for improving EF in those with IA. 

Collectively, given the possible links between PA, EF, and IA, the purpose of this pilot study was to examine whether a strategic basketball-based PA program can improve EF, specifically, cognitive flexibility evaluated by task switching, in children with IA. We hypothesized that cognitive flexibility and ball skills would be improved after the basketball PA intervention. To the best of our knowledge, this study is the first to apply a broad range of measures to examine the effect of PA on EF in children with IA. The findings of this study can pave the way for further exploration of cognitively engaging PA for ameliorating symptoms of IA by improving EF. 

## 2. Materials and Methods

### 2.1. Participants

The participants were recruited through an advertisement placed at a local elementary school in Taipei city. Eligible participants were ten school-aged children (one female; age range: 9–11 years; mean age = 10.45 ± 0.68 years) and identified using the Chen Internet Addiction Scale (CIAS) [25]. Before starting the testing, the children’s legal guardians completed questionnaires on their health history and physical activity readiness (Physical Activity Readiness Questionnaire). For inclusion in this study, participants had to meet the following criteria: (a) age between 8 and 12 years; (b) normal or corrected-to-normal vision; (c) no physical disabilities that could be exacerbated by performing exercise; (d) no doctor-diagnosed neurological or attention disorders (e.g., DCD, ADHD); (e) no comorbid developmental disorders, including learning difficulties, dyslexia, Tourette’s syndrome, epilepsy, and pervasive developmental disorder. All participants provided written informed assent, and their legal guardians provided written informed consent following the rules of the Institutional Review Board of National Taiwan University (IRB reference number: 201710HM024).

### 2.2. Measures

#### 2.2.1. Motor Competence Assessment

The participants’ motor competence was assessed using the Movement Assessment Battery for Children-2 (MABC-2), a standardized test for the identification of children with motor difficulties. The MABC-2 was designed to evaluate different types of motor competence, namely manual dexterity (placing pegs, threading a lace, and drawing trials), ball skills (two-hand catch and throwing a beanbag onto a mat), and static and dynamic balance (one-board balance, walking heel-to-toe forward, and hopping on mats), in children between 3–16 years of age. Age-adjusted standardized scores for the subscales and the overall composite score are reported, with higher scores indicating superior motor competence. The reliability coefficients were 0.80 for the composite score and from 0.73 to 0.84 for the subscale scores [26].

#### 2.2.2. Cognitive Task 

We assessed cognitive performance by using a modified task-switching paradigm [12], including a pure and a mixed task condition and three subtests. In the task, a numeral (digits 1–9, excluding 5) was presented in white text in the center of the computer screen on a black background. Pure conditions include the first and second subtests. For the first subtest, the participants had to identify whether the number surrounded by a solid-line rectangle was bigger or smaller than 5 (i.e., AAA…). In the second subtest, they had to identify whether the number surrounded by a dashed square was odd or even (i.e., BBB…). For the mixed condition, the third subtest combined the two pure conditions for an alternating-runs paradigm (i.e., AABBAA…). Digits were presented for 400 ms with a 2500 ms response-stimulus interval. If no response was made, the trial terminated 3500 ms after the onset of the stimulus. The participants were instructed to use the thumb of each hand to make a corresponding response on the keypad as quickly and accurately as possible. Participants completed 64 trials in each of the pure task conditions and 128 trials (64 trials × 2 blocks) in the mixed-task condition. The viewing distance was approximately 60 cm, and visual angles were 3.82°. Task performance was measured in terms of reaction times (RTs) and response accuracy in pure, mixed, non-switching, and switch trials. The effects of global (difference in RTs between the pure and mixed blocks) and local (difference in RTs between the non-switch and switch trials in the mixed blocks) switch costs on RT were calculated.

### 2.3. Procedure

Participants’ task switching and motor competence were assessed before and after a 12-week strategic PA program (twice per week, 90 min per session). In the pretest stage, the participants and their parents provided informed consent and the child’s health history and demographic information. Participants’ cognitive task performance and motor competence were assessed. During the intervention stage, participants participated in a strategic PA program twice a week after school for 12 weeks. In the post-test, the participants returned to the laboratory within a week at the end of the intervention and completed the same cognitive task and motor competence test as in the pretest. The participants and their parents were debriefed on the purpose and expectations of the present study after the completion of all of the experimental activities.

### 2.4. Strategic PA Program

The strategic PA program, a basketball activity, took place over 12 consecutive weeks in the sports center of an elementary school. It was held twice a week for 90 min, and each session consisted of 5 min of warmup, 20 min of fitness skills practice, 40 min of fundamental skills practice, 20 min of game set activity, and 5 min of cool down. The PA program was chosen based on physical activity guidelines for school-aged children [27] and a protocol developed for children with attention-deficit/hyperactivity disorder [28]. In the warmup and fitness skills practice, the activities combined stretching and fitness skills training such as agility, coordination, speed, and reaction time activities. The individual skills training included dribbling, passing and catching, lay-ups, and shooting drills. The game was designed to integrate the performance of individual skills with the concept of the game. This exercise provided an opportunity for the participants to refine their motor skills and increase aerobic capacity.

### 2.5. Data and Statistical Analysis

To assess motor competence, manual dexterity, ball skills, balance, and overall composite score components were separately analyzed, and the Wilcoxon signed-rank test was used to compare the pretest versus post-test scores for each MABC-2 subtest. To assess cognitive task performance, the effect of the PA program on task-switching from the pre- to the post-test was examined by using the Wilcoxon signed-rank test to compare RT and response accuracy in pure, mixed, non-switching, and switch trials, and global and local switch costs of RT separately. The Wilcoxon signed-rank test was used for within-group comparison because of the small sample size and violations of normality. Effect sizes were calculated manually using the equation: *r* = z/(n). An α level of 0.05 was used as the level of statistical significance for all statistical analyses. 

## 3. Results

### 3.1. Participant Characteristics

The participants consisted of nine boys and one girl. The body mass index (BMI) score was 17.61. Descriptive characteristics of participants are presented in Table 1.

### 3.2. Motor Competence Assessment

Table 2 summarizes the participants’ motor competence scores (mean and SD). In the total and subtest of the MABC-2, the Wilcoxon signed-rank test comparison revealed a significant improvement in manual dexterity (pre = 10.90 ± 2.18 vs. post = 12.40 ± 2.50, *p* = 0.04) and total score (pre = 9.90 ± 2.51 vs. post = 11.30 ± 2.31, *p* = 0.04) from the pre- to the posttest. However, there were no significant differences in ball skills (pre = 8.80 ± 3.58 vs. post = 10.20 ± 2.49, *p* = 0.12), or static and dynamic balance (pre = 10.20 ± 2.49 vs. post = 9.90 ± 2.56, *p =* 0.34).

### 3.3. Cognitive Task Performance

Table 3 summarizes the behavioral data for the four task-switching trials across the pre- and post-test.

#### 3.3.1. Reaction Time

Regarding reaction time, the Wilcoxon signed-rank test comparisons revealed no significant differences between pre- and post-test RT in the pure (pre = 751.20 ± 105.68 ms vs. post = 766.68 ± 155.59 ms, *p* = 0.48), mixed (pre = 1142.12 ± 191.72 ms vs. post = 1211.36 ± 237.39 ms, *p* = 0.14), non-switch (pre = 1120.65 ± 204.98 ms vs. post = 1205.59 ± 249.15, *p* = 0.08), and switch (pre = 1163.74 ± 189.31 ms vs. post = 1217.18 ± 236.99 ms, *p* = 0.22) trials.

#### 3.3.2. Global and Local Switch Costs

Regarding global and local switch costs of RT, there were no significant differences between the pre- and post-test measures in the global switch costs (pre = 390.92 ± 154.53 ms vs. post = 444.68 ± 135.94 ms, *p* = 0.10) and local switch costs (pre = 43.09 ± 92.36 ms vs. post = 11.59 ± 97.91 ms, *p* = 0.19).

#### 3.3.3. Response Accuracy

Regarding response accuracy, the Wilcoxon signed-rank test comparisons revealed significant improvement from pretest to posttest in the pure (pre = 87.10 ± 8.52% vs. post = 95.70 ± 2.95%, *p* = 0.01), mixed (pre = 70.30 ± 14.05% vs. post = 81.10 ± 10.06%, *p* = 0.02), non-switch (pre = 71.00 ± 13.81% vs. post =79.30 ± 10.73%, *p* = 0.02), and switch (pre = 69.80 ± 14.85% vs. post = 82.70 ±10.09%, *p* = 0.01) trials. 

## 4. Discussion

The current findings suggest that 12 weeks of a strategic PA program improved executive function in children with IA. Furthermore, this general facilitation was reflected in both motor competence and response accuracy in the cognitive task. This study was, to the best of our knowledge, the first to examine the effect of a strategic PA program on cognitive flexibility in children with IA. This investigation provides initial evidence that strategic PA programs can be a treatment for children with IA.

It is important to note that our results show significant increases in manual dexterity and total score after the intervention. The improved scores for manual dexterity and total score were expected because the fundamental skills that are required in basketball include movement skills, such as passing and catching, throwing and shooting, and social skills, such as communication and cooperation, all of which were included in the program. Moreover, basketball emphasizes quick responses on the court through physical activities and physical skills, as confirmed by the posttest of manual dexterity and total score. A previous study indicated that the main factors contributing to children’s physical and motor fitness while playing basketball were physical and coordination abilities (68%), throwing accuracy, and endurance (10%) [29]. Specifically, our strategic PA program included actual games, the participants had plenty of opportunities to practice different ways of passing, including chest passes and bounce passes with one or both hands, and catching the ball. Shooting practice helped the participants to gradually improve their bank shot and jump shot accuracy, and the motivation to win also encouraged the participants to improve their shooting skills during the games. Therefore, this basketball program focused on practicing various physical skills related to ball handling, dribbling, passing, catching, and shooting, suggesting that the protocol was effective in the development of motor skills.

Notably, our study observed an improvement in the accuracy of task-switching performance after the strategic PA program intervention. However, no differences were observed in RTs. The task-switching paradigm has been used extensively to examine EF, particularly aspects of working memory, inhibition, and mental flexibility. The main finding of this study is that the strategic sports program improved accuracy performance on pure, mixed, switch, and no-switch trials. These results suggest that practicing a long-term strategy sport not only influences EF in general but also may improve EF in children with IA. These findings may be particularly important because individuals with IA often exhibit impairment in EF, particularly inhibition [30] and response monitoring [31]. Furthermore, a previous study indicated that control processes are particularly impaired when individuals with IA [8]. There is growing evidence suggesting that cognitive and motor functions are not only interrelated but also may rely on the development of the same cortical and subcortical neural structures [15]. The parallel development of these neural structures may explain the close association between motor competence and EF found in typically developing children [32] and children with ADHD [21]. In addition to the neurophysiological explanations, a previous study indicated that the beneficial effect of sports participation on alleviating symptoms of IA was mediated by self-control [33]. Physical activities rich in motor skills, such as the basketball program used in the present study, are particularly effective in ameliorating the EF deficit in children with IA. This psychological explanation also provides another possible explanation for why physical activity can benefit EF in children with IA. 

Despite the current results revealing no training effect on RT and the lack of a fa-cilitatory effect on global and local switch costs, which is consistent with previous re-search [34]. from a speed–accuracy trade-off perspective, we might speculate that the strategic sports program helped participants to slow down their responses to ensure that they were responding accurately. Thus, adopting a strategy of slower performance and being able to detect when it is advantageous to do so may be one mechanism for improving accuracy in set shifting [35].

## 5. Conclusions

In conclusion, the present study highlights the positive effect of a strategic PA program on EF in children with IA. Specifically, the use of the task-switching paradigm suggests that long-term strategic PA programs might facilitate cognitive flexibility, which is responsible for the core dysfunctions in children with IA. However, there are several limitations to the current study that may inform future research directions. First, due to the difficulty in recruiting children with IA, the small sample size may have been somewhat underpowered for detecting significant differences. Therefore, the results reported are preliminary and should be interpreted with caution. Second, the single group pretest-posttest design made it difficult to draw causal inferences. However, the findings can be treated as a reference for future longitudinal or interventional studies. Such studies should include a control group, preferably with a randomized controlled trial design, to validate the findings of the present study. Further research on understanding how long-term strategic PA programs influence EF processes in children with IA is warranted. Third, given our primary interest in EF and motor abilities, future studies would benefit from the use of additional neurocognitive measures (e.g., electroencephalography, functional magnetic resonance imaging) to assess specific aspects of EF and more detailed assessments of motor skills and coordination.

## Figures and Tables

**Table 1:** Participants’ Demographic Characteristics.

Demographic Variables	Mean ± SD
Gender (Male:Female)	9:1
Age (years)	10.45 ± 0.68
Height (cm)	143.51 ± 5.81
Weight (kg)	36.68 ± 7.68
BMI (kg/m^2^)	17.61 ± 2.57

Note: BMI = body mass index.

**Table 2:** Participants’ Motor Competence Scores.

MABC-2 Variables	Pretest	Posttest	*p*-Value	*r*
	Mean ± SD	Mean ± SD		
Manual dexterity	10.90 ± 2.18	12.40 ± 2.50	0.04	−0.40
Ball skills	8.80 ± 3.58	10.20 ± 2.49	0.12	−0.26
Balance	10.20 ± 2.49	9.90 ± 2.56	0.34	−0.10
Total	9.90 ± 2.51	11.30 ± 2.31	0.04	−0.38

**Table 3:** Switching Task Reaction Times and Accuracy.

Variable	Pretest	Posttest	*p*-Value	*r*
	Mean ± SD	Mean ± SD		
RT (ms)				
Pure trials	751.20 ± 105.68	766.68 ± 155.59	0.48	−0.01
Mixed trials	1142.12 ± 191.72	1211.36 ± 237.39	0.14	−0.24
Non-switch trials	1120.65 ± 204.98	1205.59 ± 249.15	0.08	−0.31
Switch trials	1163.74 ± 189.31	1217.18 ± 236.99	0.22	−0.17
Global switch cost	390.92 ± 154.53	444.68 ± 135.94	0.10	−0.28
Local switch cost	43.09 ± 92.36	11.59 ± 97.91	0.19	−0.19
Accuracy (%)				
Pure trials	87.10 ± 8.52	95.70 ± 2.95	0.01	−0.57
Mixed trials	70.30 ± 14.05	81.10 ± 10.06	0.02	−0.48
Non-switch trials	71.00 ± 13.81	79.30 ± 10.73	0.02	−0.44
Switch trials	69.80 ± 14.85	82.70 ±10.09	0.01	−0.51

## Data Availability

The data presented in this study are available from the corresponding author on reasonable request.

## References

[1] Saunders J.B. (2017). Substance use and addictive disorders in DSM-5 and ICD 10 and the draft ICD 11. Curr. Opin. Psychiatry.

[2] Hechanova R.M., Czincz J. (2008). Internet addiction in Asia: Reality or myth?. Int. Dev. Res. Cent. Digit. Libr..

[3] Lan C.M., Lee Y.H. (2013). The predictors of internet addiction behaviours for Taiwanese elementary school students. Sch. Psychol. Int..

[4] Lin M.-P., Wu J.Y.-W., You J., Hu W.-H., Yen C.-F. (2018). Prevalence of internet addiction and its risk and protective factors in a representative sample of senior high school students in Taiwan. J. Adolesc..

[5] Kuss D.J., Griffiths M.G., Karila L., Billieux J. (2014). Internet addiction: A systematic review of epidemiological research for the last decade. Curr. Pharm. Des..

[6] Winkler A., Dörsing B., Rief W., Shen Y., Glombiewski J.A. (2013). Treatment of internet addiction: A meta-analysis. Clin. Psychol. Rev..

[7] Friedman N.P., Miyake A., Altamirano L.J., Corley R.P., Young S.E., Rhea S.A., Hewitt J.K. (2016). Stability and change in executive function abilities from late adolescence to early adulthood: A longitudinal twin study. Dev. Psychol..

[8] Brand M., Young K.S., Laier C. (2014). Prefrontal control and Internet addiction: A theoretical model and review of neuropsychological and neuroimaging findings. Front. Hum. Neurosci..

[9] Ioannidis K., Hook R., Goudriaan A.E., Vlies S., Fineberg N.A., Grant J.E., Chamberlain S.R. (2019). Cognitive deficits in problematic internet use: Meta-analysis of 40 studies. Br. J. Psychiatry.

[10] Dong G., Lin X., Zhou H., Lu Q. (2014). Cognitive flexibility in internet addicts: fMRI evidence from difficult-to-easy and easy-to-difficult switching situations. Addict. Behav..

[11] Sibley B.A., Etnier J.L. (2003). The relationship between physical activity and cognition in children: A meta-analysis. Pediatric Exerc. Sci..

[12] Hung C.L., Huang C.J., Tsai Y.J., Chang Y.K., Hung T.M. (2016). Neuroelectric and behavioral effects of acute exercise on task switching in children with attention-deficit/hyperactivity disorder. Front. Psychol..

[13] Chang Y.K., Hung C.L., Huang C.J., Hatfield B.D., Hung T.M. (2014). Effects of an aquatic exercise program on inhibitory control in children with ADHD: A preliminary study. Arch. Clin. Neuropsychol..

[14] Hillman C.H., Pontifex M.B., Castelli D.M., Khan N.A., Raine L.B., Scudder M.R., Drollette E.S., Moore R.D., Wu C.-T., Kamijo K. (2014). Effects of the FITKids randomized controlled trial on executive control and brain function. Pediatrics.

[15] Diamond A. (2000). Close interrelation of motor development and cognitive development and of the cerebellum and prefrontal cortex. Child Dev..

[16] Han G., Zhang J., Ma S., Lu R., Duan J., Song Y., Lau P.W.C. (2021). Prevalence of internet addiction and its relationship with combinations of physical activity and screen-based sedentary behavior among adolescents in China. J. Phys. Act. Health.

[17] Nguyen P., Le L.K.-D., Nguyen D., Gao L., Dunstan D.W., Moodie M. (2020). The effectiveness of sedentary behaviour interventions on sitting time and screen time in children and adults: An umbrella review of systematic reviews. Int. J. Behav. Nutr. Phys. Act..

[18] Law L.L., Barnett F., Yau M.K., Gray M.A. (2014). Effects of combined cognitive and exercise interventions on cognition in older adults with and without cognitive impairment: A systematic review. Ageing Res. Rev..

[19] Singer R.N. (2000). Performance and human factors: Considerations about cognition and attention for self-paced and externally-paced events. Ergonomics.

[20] Roebers C.M., Kauer M. (2009). Motor and cognitive control in a normative sample of 7-year-olds. Dev. Sci..

[21] Hung C.L., Chang Y.K., Chan Y.S., Shih C.H., Huang C.J., Hung T.M. (2013). Motor ability and inhibitory processes in children with ADHD: A neuroelectric study. J. Sport Exerc. Psychol..

[22] Turkeri C., Ozturk B., Buyuktas B., Ozturk D. (2019). Comparison of balance, reaction time, attention and BMI values on individual and team sports. J. Educ. Learn..

[23] Policastro F., Accardo A., Marcovich R., Pelamatti G., Zoia S. (2018). Relation between motor and cognitive skills in Italian basketball players aged between 7 and 10 years old. Sports.

[24] Policastro F., Accardo A., MArcovich R., Pelamatti G., Zoia S. (2019). Correlations between motor and cognitive skills in young basketball players: A bivariate regression analysis. Phys. Med. Rehabil. Res..

[25] Chen S.-H., Weng L.-J., Su Y.-J., Wu H.-M., Yang P.-F. (2003). Development of a Chinese Internet addiction scale and its psychometric study. Chin. J. Psychol..

[26] Henderson S., Sugden D., Barnett A. (2007). Movement Assessment Battery for Children.

[27] Janssen I., Le Blanc A.G. (2010). Systematic review of the health benefits of physical activity and fitness in school-aged children and youth. Int. J. Behav. Nutr. Phys..

[28] Pan C.Y., Tsai C.L., Chu C.H., Sung M.C., Huang C.Y., Ma W.Y. (2015). Effects of physical exercise intervention on motor skills and executive functions in children with ADHD: A pilot study. J. Atten. Disord..

[29] Andrieieva O., Yarmak O., Kyrychenko V., Ravliuk T., Tsurkan T., Zavgorodnia T., Strazhnikova I., Potop V. (2020). The factor structure of physical and motor fitness of 12-year-old children while playing basketball. J. Phys. Educ. Sport.

[30] Dong G., Lu Q., Zhou H., Zhao X. (2010). Impulse inhibition in people with Internet addiction disorder: Electrophysiological evidence from a Go/NoGo study. Neurosci. Lett..

[31] Zhou Z., Li C., Zhu H. (2015). An error-related negativity potential investigation of response monitoring function in individuals with Internet addiction disorder. Front. Behav. Neurosci..

[32] Haapala E.A., Poikkeus A.-M., Tompuri T., Katriina K.-H., Leppänen P., Lindi V., Lakka T.A. (2013). Associations of motor and cardiovascular performance with academic skills in children. Med. Sci. Sports Exerc..

[33] Park J.-A., Park M.-H., Shin J.-H., Li B., Rolfe D.T., Yoo J.-Y., Dittmore S.W. (2016). Effect of sports participation on internet addiction mediated by self-control: A case of korean adolescents. Kasetsart J. Soc. Sci..

[34] Hsieh S.S., Lin C.C., Chang Y.K., Huang C.J., Hung T.M. (2017). Effects of childhood gymnastics program on spatial working memory. Med. Sci. Sports Exerc..

[35] Davidson M.C., Amso D., Anderson L.C., Diamond A. (2006). Development of cognitive control and executive functions from 4 to 13 years: Evidence from manipulations of memory, inhibition, and task switching. Neuropsychologia.

